# Physical–Chemical Assessment and Antimicrobial Activity of Chlortetracycline-Loaded Collagen Sponges

**DOI:** 10.3390/ma18174029

**Published:** 2025-08-28

**Authors:** Graţiela Teodora Tihan, Camelia Ungureanu, Ileana Rău, Roxana Gabriela Zgârian, Răzvan Constantin Barbaresso, Mădălina Georgiana Albu Kaya, Cristina-Elena Dinu-Pîrvu, Mihaela Violeta Ghica

**Affiliations:** 1 Department of General Chemistry, Faculty of Chemical Engineering and Biotechnology, National University of Science and Technology Politehnica of Bucharest, 1-7 Gheorghe Polizu Street, 011061 Bucharest, Romania; gratielatihan@yahoo.com (G.T.T.); ungureanucamelia@gmail.com (C.U.); ileana.rau@upb.ro (I.R.); razvanb67@yahoo.com (R.C.B.); 2 Department of Collagen, Division of Leather and Footwear Research Institute, National Research and Development Institute for Textiles and Leather, 93 Ion Minulescu Str., 031215 Bucharest, Romania; albu_mada@yahoo.com; 3 Department of Physical and Colloidal Chemistry, Faculty of Pharmacy, “Carol Davila” University of Medicine and Pharmacy, 6 Traian Vuia Street, 020956 Bucharest, Romania; ecristinaparvu@yahoo.com (C.-E.D.-P.); mihaelaghica@yahoo.com (M.V.G.); 4 Innovative Therapeutic Structures Research and Development Center (InnoTher), “Carol Davila” University of Medicine and Pharmacy, 6 Traian Vuia Street, 020956 Bucharest, Romania

**Keywords:** collagen, chlortetracycline, drug release, antimicrobial activity

## Abstract

Collagen-based biomaterials are increasingly explored in dentistry for their ability to deliver drugs locally and support healing. In this study, we developed chlortetracycline-loaded collagen sponges aimed at preventing postoperative infections. Five formulations were prepared by lyophilization, each with the same collagen-to-drug ratio but different glutaraldehyde (GA) concentrations: 0%, 0.25%, 0.5%, 0.75%, and 1% (*w*/*w*) relative to dry collagen. The sponges were characterized using FT-IR and UV–VIS–NIR spectroscopy, and their swelling capacity, enzymatic stability, and drug release kinetics were evaluated. Antibacterial activity was tested against *Escherichia coli*, *Staphylococcus aureus*, and *Enterococcus faecalis*. Statistical differences between formulations were assessed using one-way ANOVA followed by Tukey’s post hoc test (*p* < 0.05). All sponges released the antibiotic rapidly within the first 60 min, followed by a sustained release for up to 10 h. The non-crosslinked sponge showed the highest antimicrobial effect, while the 0.25% GA formulation offered a good balance between stability and bioactivity. While higher cross-linking enhanced structural stability, it progressively reduced antimicrobial efficacy, highlighting a crucial design trade-off. These findings underline the need to fine-tune cross-linking conditions to achieve both durability and strong antimicrobial action in collagen-based drug delivery systems for dental applications.

## 1. Introduction

Modern dental materials are increasingly designed not only to restore or replace damaged tissues, but also to support healing and reduce the risk of infection [1,2,3,4]. Among these, collagen-based composites stand out for their excellent biocompatibility, biodegradability, and their ability to act as carriers for bioactive compounds [5,6,7]. One of the main challenges in periodontal and endodontic procedures remains the risk of bacterial colonization and biofilm formation, which can significantly compromise the success of restorative interventions [8,9,10,11,12]. To address this issue, collagen scaffolds enriched with antimicrobial agents such as chlortetracycline have emerged as promising strategies for local infection control. Evaluating their antimicrobial performance is crucial both for confirming therapeutic efficacy and for optimizing their composition for clinical use.

Furthermore, collagen-based sponges and scaffolds have long been recognized for their capacity to actively support tissue healing. Beyond serving as inert carriers, collagen matrices mimic the extracellular matrix, facilitate cell adhesion and proliferation, and accelerate soft tissue regeneration in oral surgery and tooth extraction sites [13,14]. A randomized clinical study demonstrated that inserting a collagen sponge into extraction sockets significantly reduced early postoperative complications and enhanced periodontal healing [15]. Collagen matrices have also been employed for the local delivery of anesthetics in dental procedures, further highlighting their versatility and clinical relevance [16].

Traditional intracanal disinfection methods, such as calcium hydroxide or sodium hypochlorite irrigation, remain standard in endodontics. However, they exhibit limitations: sodium hypochlorite, despite its potent antimicrobial effects, can be cytotoxic and may interfere with bonding agents; calcium hydroxide shows limited efficacy against key pathogens such as *E. faecalis*, has slow action, and its high alkalinity may compromise dentin integrity [17,18]. Moreover, biofilm-associated bacteria can exhibit vastly reduced sensitivity to antimicrobials—up to 1000–1500 times less than planktonic cells—making alternative strategies necessary [19].

In this light, the development of collagen scaffolds capable of delivering antimicrobial agents provides an attractive route: combining the material’s intrinsic regenerative properties with localized, sustained drug release [20]. Early-stage research into collagen-based delivery systems across wound care and dental applications has highlighted the versatility of collagen formats—ranging from hydrogels and films to sponges—as effective platforms for antimicrobial agent integration [13,20].

Our current study builds on this foundation by formulating chlortetracycline-loaded collagen sponges via lyophilization, aiming to blend the biocompatibility and structural support inherent to collagen with precise antibiotic delivery. This integration addresses both the need for prolonged local antimicrobial action and the shortcomings of conventional disinfectants, particularly in challenging environments such as root canal biofilms. Given the renewed interest in reformulating well-characterized, older-generation antibiotics, chlortetracycline was selected for its reliable antibacterial performance and suitability for incorporation into collagen-based scaffolds [21,22,23]. In addition to its well-established antimicrobial spectrum, chlortetracycline was selected for its compatibility with collagen-based matrices, its availability in topical pharmaceutical formulations, and its renewed interest in the context of localized therapies targeting antibiotic-resistant pathogens [22,23].

In this study, the antimicrobial activity of chlortetracycline-loaded collagen scaffolds was tested against three bacterial strains commonly associated with oral infections and treatment failure. *Escherichia coli* (*E. coli*) is a lactose-positive, Gram-negative, oxidase-negative bacillus from the Enterobacteriaceae family. Although not typically present in healthy root canals, it has been identified in necrotic pulp and infected periapical lesions in certain clinical cases. Its inclusion in dental research is justified by its potential to indicate fecal contamination and its relevance as a Gram-negative model organism for testing antimicrobial biomaterials [24,25,26]. *Enterococcus faecalis* (*E. faecalis*) is a facultative anaerobic, Gram-positive coccus well known for its ability to survive in harsh environments and for its intrinsic multidrug resistance [27]. It is one of the most persistent pathogens encountered in endodontics, often isolated from failed root canal treatments. Its capacity to penetrate dentinal tubules and resist intracanal medications makes it a benchmark microorganism for evaluating root canal disinfection protocols [28,29,30,31,32]. *Staphylococcus aureus* (*S. aureus*) is a Gram-positive, coagulase-positive bacterium responsible for a wide range of infections, from superficial lesions to severe systemic conditions. In dentistry, it is often associated with post-operative infections following implantology or prosthetic interventions. Due to its high virulence and resistance mechanisms, *S. aureus* is considered a critical target when evaluating antimicrobial performance of dental materials [33,34,35].

The development of spongy collagen-based biomaterials responds to the growing need for advanced therapeutic solutions in dental care. Antibiotics are frequently incorporated into such materials for their broad-spectrum antimicrobial activity [36,37,38,39]. In our previous studies, we developed and characterized various collagen-based systems containing antibiotics [10,40] or anti-inflammatory agents [8,41], with the goal of enhancing their therapeutic potential. These formulations have shown promising bacteriostatic activity against both Gram-positive and Gram-negative bacteria.

Building upon this foundation, we further investigated chlortetracycline—a first-generation tetracycline-class antibiotic [21]—which is currently regaining scientific interest due to its well-documented pharmacological profile and known safety record [22]. Presently, chlortetracycline is used in various commercial pharmaceutical forms for ophthalmic (e.g., Chlortralim 1%, ChlotraCare 0.1% and 1%, Ophtocycline 10 mg/g) and cutaneous applications (e.g., Aureomicina 3%, Aureocort—a combination with triamcinolone acetonide) [42,43,44,45,46]. Its potential in dentistry has also been highlighted in several studies [47,48,49], particularly in systems enabling localized antibiotic delivery [23].

Moreover, Ledermix—a paste containing demeclocycline calcium (a derivative of chlortetracycline) and triamcinolone acetonide—remains the only clinically validated product used successfully in root canal treatments. Its application as an intracanal medication helps reduce postoperative pain, inflammation, and bacterial load [50]. These findings support the selection of chlortetracycline as a suitable antibiotic for developing dental biomaterials with localized action.

Building on its well-documented safety and broad-spectrum antimicrobial profile, chlortetracycline was selected as the active agent for incorporation into collagen sponges intended for local delivery in dental applications. While previous studies have explored collagen–antibiotic systems, few have systematically investigated how varying the degree of glutaraldehyde cross-linking influences both drug release kinetics and antimicrobial performance. This aspect is particularly relevant given the inherent trade-off between enhancing structural stability and maintaining antibacterial efficacy. The present work addresses this gap by developing and characterizing five chlortetracycline-loaded collagen sponge formulations with increasing GA concentrations (0%, 0.25%, 0.5%, 0.75%, and 1% (*w*/*w*) relative to dry collagen), evaluating their physicochemical properties, release behavior, and antibacterial activity against clinically relevant oral pathogens.

## 2. Materials and Methods

### 2.1. Materials

**Figure 1:**
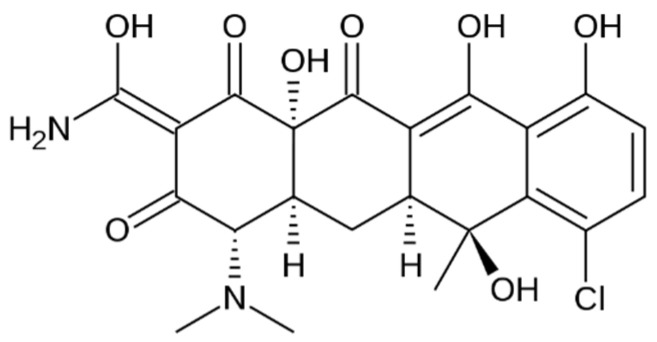
Chemical structures of CL.

Type I collagen in the form of gel (CG) was extracted from calf derma in the Department of Collagen Research from INCDTP—Division Leather and Footwear Research Institute, Romania, according with in-house technology [51]. The concentration of collagen gel obtained was 2.11%, pH = 2.5. The cross-linking agent, glutaraldehyde (GA), was purchased from Merck (Darmstadt, Germany), and the active compound, chlortetracycline HCL (CL) (Figure 1) (a first-generation tetracycline antibiotic), sourced from Delos Impex, Otopeni, Romania, batch FC08011019. For pH adjustment, under mechanical stirring, a 1 M solution of sodium hydroxide (NaOH) of analytical purity was used. For enzymatic degradation evaluation, bacterial collagenase from *Clostridium histolyticum* from Sigma-Aldrich (St. Louis, MO, USA) was used.

### 2.2. Preparation of Chlortetracycline-Loaded Collagen Sponges

In the first step, collagen gel with initial concentration of 2.11% and pH 2.5 was adjusted to 1%, and the pH to 7.4, using 1 M NaOH solution and purified water. The pH was measured using an Innolab pH-meter (WTW, Weilheim, Germany), calibrated with pH 4.00/7.00/10.00 buffers prior to measurements. It should be noted that the oral environment is dynamic and subject to pH fluctuations, especially during infection or dietary intake. Although the present study assessed sponge stability and drug release at neutral pH (7.4), future investigations should consider evaluating these parameters across a range of pH values to better simulate in vivo conditions. The pH adjustment is also essential to stabilize chlortetracycline during incorporation and to optimize the cross-linking reaction between collagen and glutaraldehyde, which is more efficient in near-neutral conditions. Chlortetracycline was incorporated into all prepared gels at a concentration of 0.1% (*w*/*v*) relative to the collagen gel content. The GA concentrations reported to the dry substance of collagen were 0, 0.25, 0.5, 0.75, and 1%. The prepared homogeneous gels were lyophilized using a Delta 2-24 LSC lyophilizer (Martin Christ, Osterode am Harz, Germany), and collagen sponges were obtained. The five chlortetracycline-loaded collagen sponges were named as follows: one sponge based on CG and CL (CG-CL), and four sponges containing CG, CL, and different GA concentrations (CG-CL GA 0.25%; CG-CL GA 0.5%; CG-CL GA 0.75%; CG-CL GA 1%). In the final step, all the sponges were packed in polyethylene bags, sterilized under UV light at 254 nm with Vilber-Lourmat equipment (Vilber-Lourmat, Collégien, France), and then stored at room temperature, at a humidity between 40% and 50% before being characterized.

A limitation of this study is the absence of data regarding the actual loading efficiency of chlortetracycline within the collagen matrix. Although the preparation protocol ensured a consistent drug-to-polymer ratio, experimental quantification of the incorporated antibiotic was not performed at the time. Unfortunately, due to the perishable nature of the samples, as well as the lack of remaining material, it was not possible to carry out this determination retrospectively.

This limitation does not affect the interpretation of the release and antimicrobial behavior discussed, but future studies should include this quantification to enhance comparability and reproducibility.

### 2.3. Experimental Techniques

The FT-IR study was carried out using a Perkin Elmer Spectrum 100 FT-IR spectrophotometer (PerkinElmer, Waltham, MA, USA) in attenuated total reflection (ATR) mode. The spectra were recorded as the average of 4 acquisitions per sample with 4 cm^−1^ resolution over the range between 4000 cm^−1^ and 600 cm^−1^. Prior to analysis, the freeze-dried sponges were visually inspected to confirm structural homogeneity across top, inner, and bottom regions. For FT-IR measurements, a fragment (~5 mm in diameter) from the upper surface was placed directly on the ATR crystal, and gentle pressure was applied to ensure optimal contact and signal quality.

UV–VIS–NIR spectroscopy was performed with a Jasco UV–VIS–NIR spectrophotometer (Jasco Inc., Tokyo, Japan), model V 670, between 200 nm and 2000 nm, with a step of 0.5 nm. For UV–VIS–NIR measurements, a ~1 cm × 1 cm sponge fragment was cut, with the upper surface oriented toward the incident beam.

For the swelling test, cylindrical samples (5 mm diameter × 10 mm thickness) were manually cut from the freeze-dried sponges using scissors, with dimensions checked by a precision ruler to ensure uniformity. Measurements were performed in triplicate for each formulation. These were weighed at the initial time (W_d_) and then immersed in distilled water at 25 °C for various intervals until a stable mass (W_w_) was reached. Equation (1) was used to calculate the water absorption W (%). The test was performed in triplicate.

% Water absorption = [(W_w_ − W_d_)/W_d_] × 100 (1)

To evaluate the structural stability, in vitro enzymatic degradation was performed using bacterial collagenase (*Clostridium histolyticum*, Sigma-Aldrich, St. Louis, MO, USA) at 37 °C, 10^−6^ g/L enzyme concentration, in phosphate-buffered saline (PBS) at pH 7.4. The weight loss % was calculated with Equation (2) [52,53], where W_i_ represents the initial weight, and W_t_ represents the weight after time t. The in vitro enzymatic degradation test was performed in triplicate.

% Weight loss = [(W_i_ − W_t_)/W_i_] × 100 (2)

The in vitro antibiotic release form collagen-based sponges were performed using a paddle dissolution apparatus (Essa dissolver, Milan, Italy), as we described in our previous research [10]. Briefly, the sponges loaded with chlortetracycline were placed in the sandwich devices and immersed in the release medium (phosphate buffer pH 7.4), at 37 °C and 50 rpm. At specific time intervals, aliquots of 5 mL of the release medium were withdrawn from the vessels and replaced by an equal volume of new phosphate buffer solution, also preheated at 37 °C. The chlortetracycline concentration released from every collagen-based sponge was assessed by a UV–VIS spectrophotometer.

To evaluate the mechanism of the antibiotic delivery from collagen sponges, the recorded data were fitted with the Power law model and one particular case, Higuchi (Equation (3)):

(3) $$\frac{M_{t}}{M_{\infty }}={kt}^{n}$$

where M_t_/M_∞_ is the fractional antibiotic release at time t, k is the kinetic constant associated with the drug–biopolymer support (1/min^n^), and n is the release exponent specific to the drug release mechanism (dimensionless).

For the statistical analysis, GraphPad Prism 10.3.1 software was used.

The antimicrobial activity of the collagen-based composites was evaluated using a liquid-phase assay. The active compound used was chlortetracycline HCl (a first-generation tetracycline antibiotic) sourced from Delos Impex, Romania, batch FC08011019. Tetracyclines exhibit broad-spectrum antimicrobial activity against both Gram-positive and Gram-negative bacteria. Generally, Gram-positive organisms are inhibited at lower tetracycline concentrations compared to Gram-negative ones [54,55]. Tetracyclines are also active against most spirochetes and a wide range of anaerobic bacteria, making them relevant in periodontal applications. Moreover, pretreatment of root surfaces with tetracyclines has been shown to enhance fibroblast attachment and colonization [56,57]. These drugs can bind to dentin and contribute to its demineralization. However, it remains unclear whether these effects are due to chemical alteration of dentin properties or the release of components such as collagen I, osteonectin, proteoglycans, or growth factors.

To assess antibacterial efficacy, each collagen sponge was incubated in Luria Bertani (LB) broth inoculated with a standardized bacterial suspension (0.5 McFarland standard) of the test organisms: *Staphylococcus aureus* ATCC 25923, *Escherichia coli* ATCC 11229, and *Enterococcus faecalis* ATCC 29212. Prior to testing, the strains were cultivated on a nutrient-rich LB medium composed of peptone (10 g/L), yeast extract (5 g/L), NaCl (5 g/L), and maintained at 4 °C. These strains were chosen due to their clinical relevance in dental and periodontal infections, as previously described in the Introduction, where their pathogenic potential, persistence in endodontic environments, and resistance to standard treatments are outlined.

The minimum inhibitory concentration (MIC) was determined as the lowest concentration of antibiotic capable of inhibiting visible microbial growth following 18 h of incubation at 37 °C. To establish the MIC values, a series of logarithmic dilutions ranging from 400 µg/mL to 0.195 µg/mL were prepared, inoculated with standardized microbial cultures, and monitored for bacterial growth. Because raw outcomes were S/R, MICs are reported as interval estimates (MIC ∈ (C_R, C_S]); the full S/R grid is provided in Table S1 (Supplementary Materials). Here, C denotes a tested concentration step. C_S is the first step marked S; C_R is the next lower step marked R. Thus, MIC is reported as an interval (C_R, C_S], i.e., MIC > C_R and MIC ≤ C_S.

Antimicrobial activity was expressed as a percentage inhibition relative to the positive control (bacterial growth in the absence of the collagen sponge). All measurements were conducted in triplicate to ensure statistical reliability. Briefly, quantitative evaluation of antibacterial activity was performed using a liquid-phase inhibition assay. For antibacterial activity testing, sterile collagen sponge fragments (~3 mg each) were obtained by cutting small pieces from the freeze-dried sponges and weighing them individually on an analytical balance (±0.1 mg precision). This procedure was applied uniformly across all formulations, and weight variation between samples did not exceed ±0.05 mg. Sterile collagen sponge samples were incubated for 18 h in test tubes containing 5 mL bacterial suspension at 37 °C using a Laboshake Gerhardt incubator (Konigswinter, Germany). Optical densities (ODs) of the samples and controls were measured at 600 nm using a UV–VIS spectrophotometer (Jenway, Hong Kong, China). The antibacterial effect was expressed as inhibition percentage (I%) calculated using the formula (Equation (4)):

I% = [(B_18_ − B_0_) − (C_18_ − C_0_)]/(B_18_ − B_0_) × 100 (4)

where B_18_ = OD of bacterial control at 18 h; B_0_ = OD of bacterial control at time 0; C_18_ = OD of treated sample at 18 h; and C_0_ = OD of treated sample at time 0.

For statistical analysis, all prepared sponges were evaluated in triplicate, and the results were expressed as mean ± standard deviation (SD) of replicates. The differences between means were determined using analysis of variance (one-way ANOVA) and Tukey’s test, considering significant differences for *p* values < 0.05. Statistical analyses, including one-way ANOVA and Tukey’s post-hoc test, were performed using Microsoft Excel (Microsoft 365), with the Analysis ToolPak add-in activated.

No a priori power calculation was performed. Given the exploratory nature of this study and manufacturing constraints, we used *n* = 3 technical replicates per formulation for each in-vitro assay. Data are reported as mean ± SD. Group comparisons were conducted using one-way ANOVA with Tukey’s post-hoc test (*p* < 0.05).

## 3. Results and Discussion

Collagen-based biomaterials were prepared by lyophilization (freeze-drying) of the corresponding gels to obtain the sponges. This technology of lyophilization is one of the best methods of preservation, which allows to the dried products to keep the original value of active ingredients, such as drugs, growth factors, and peptides. By using this method, the embedded active drug (chlortetracycline) into the collagen sponges is in a certain amount and is keeping its properties.

### 3.1. FT-IR Characterization of Chlortetracycline–Collagen Interactions

**Figure 2:**
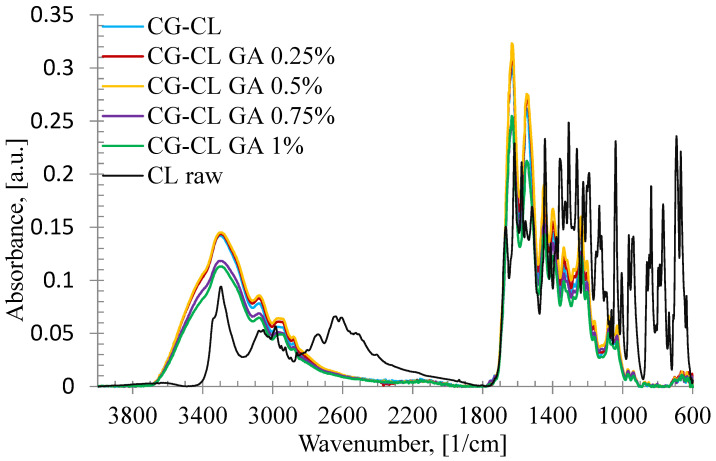
FT-IR spectra of raw CL, and CL-loaded collagen sponges.

Raw chlortetracycline, and collagen-based biomaterials prepared in the form of sponges, with the active substance incorporated, with or without the cross-linking agents, were characterized by FT-IR spectroscopy to determine the functional groups and, therefore, the structure, and the bonds formed between the raw components (Figure 2). The FT-IR spectral data of the raw collagen gel are not included here, as they have already been published in detail in our previous work [58].

In Figure 2, the IR spectrum of pure chlortetracycline shows the following specific bands: the absorption band corresponding to the stretching vibration of the O-H group (ν_OH_) found at 3296 cm^−1^; the band due to the contribution of the stretching vibration of the C=O (ν_c=o_) recorded at 1669 cm^−1^ and 1618 cm^−1^; the peak located at 1556 cm^−1^ characteristic to deformation vibration of the N-H bond (δ_N-H_); the absorption band specific to the stretching vibration of the C=C bond (ν_C=C aromatic_) registered at 1518 cm^−1^; the C-H bond characteristic to deformation vibration of the CH_3_ group (δ_CH3_) highlighted at 1329 cm^−1^; the absorption band at 1064 cm^−1^ corresponding to the stretching vibration of the C-OH bond (ν_C–OH_).

Analyzing the IR spectra of prepared collagen sponges, in all cases, the absorption bands corresponding to the pure substances as CG [58] and chlortetracycline (Figure 2) were recorded and bands specific to hydrogen bonds formed between pure substances in the presence or absence of the cross-linking agent. Thus, the absorption band attributed to the stretching vibration of O-H group (ν_OH_) in raw components and to the hydrogen bonds formed between molecules appeared at around 3300 cm^−1^. Other important vibrations recorded are specific to collagen: the stretching vibration of the C=O bond (ν_C=O_), the stretching vibration of the C-N (ν_C-N_) bond, and the deformation vibration of the C-C-N bond (δ_C-C-N_) at around 1635 cm^−1^ from Amides I; the deformation vibration of the N=H bond (δ_N-H_), and the stretching vibration of the C-N bond (ν_C-N_) at around 1550 cm^−1^ from Amide II; and the stretching vibration of the C-N bond (ν_C-N_), and also the C-C bond at around 1337 cm^−1^ from Amide III.

Moreover, from IR spectra of all collagen sponges, using different ratios between absorption band intensities, the following information [59] were obtained and collected in Table 1: the hydrolysis degree by A_OH_/A_I_ ratio, the cross-linking degree by A_I_/A_OH_ ratio, and the distortion by Δυ = υ_Ι_ − υ_ΙΙ_, where υ_Ι_ and υ_ΙΙ_ are the wavenumbers of Amide I and Amide II. For Δυ < 100, the distortion process does not occur [60].

Overall, A_OH_/A_I_ decreased slightly and A_I_/A_OH_ increased with increasing GA, consistent with enhanced cross-linking. The apparent deviation at 0.75% GA can be rationalized by (i) competition between chlortetracycline–collagen hydrogen bonding and GA-mediated cross-links at intermediate cross-linker levels, (ii) potential diffusion-limited GA access leading to local network heterogeneity during gelation/lyophilization, and (iii) the contribution of bound water to the ~3300 cm^−1^ A_OH_ band, which can subtly affect intensity ratios. Notably, Δ remained < 100, indicating no denaturation. In agreement, the in vitro release data show a monotonic decrease in cumulative drug release with increasing GA (0.25–1%), supporting that the 0.75% value reflects a minor spectral fluctuation rather than a true reversal of the cross-linking trend.

Although some minor peaks characteristic of raw chlortetracycline, particularly in the 2200–2700 cm^−1^ and 600–800 cm^−1^ ranges, were not clearly visible in the spectra of the CG-CL samples, this behavior can be explained by a combination of factors. First, the drug was incorporated at a relatively low concentration (0.1% (*w*/*v*) with respect to collagen gel content), which naturally reduces the intensity of its specific signals when dispersed in the collagen matrix.

Second, and more importantly, the chlortetracycline molecules formed hydrogen bonds with collagen chains, which can shift, broaden, or even mask their typical absorption bands. Similar spectral behavior has been reported in other polymer–drug systems, where such interactions led to the suppression or displacement of characteristic peaks [61].

These findings support the interpretation that the drug was physically entrapped within the collagen network, rather than chemically degraded during the loading process.

The degree of hydrolysis is very similar in the case of the five types of prepared sponges. It decreased very slightly with increasing the cross-linking agent concentration, and an exception occurred in the sponge with 0.75%. The values obtained from the A_I_/A_OH_ ratio indicated a high degree of cross-linking for all analyzed sponges, and the results obtained for Δυ suggest that the denaturation process was not present.

### 3.2. Optical Characterization by UV–VIS–NIR Spectroscopy

**Figure 3:**
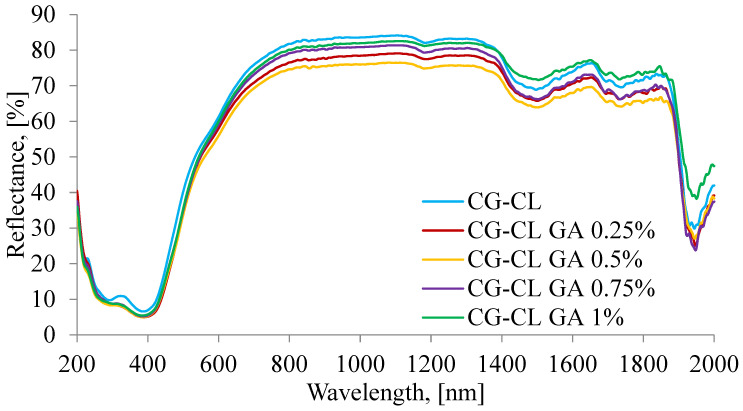
UV–VIS–NIR spectra of CL-loaded collagen sponges.

The UV–VIS–NIR spectra plotted for prepared collagen sponges are presented in Figure 3, and the spectral assignments are collected in Table 2 showing the presence of each used component and the correlation between them.

Beyond the mere presence of the bands listed in Table 2, their subtle evolution with GA is informative. The UV feature assigned to –CO–NH/CL chromophores (≈293–307 nm) exhibits a small bathochromic shift and a slight decrease in intensity as GA increases, which is consistent with stronger hydrogen bonding and reduced chromophore mobility in a denser, cross-linked network (hypochromic effect). In the NIR, the OH-associated overtone (~1497–1503 nm) and the OH combination band (~1944–1950 nm) shift to longer wavelengths by a few nanometers, pointing to an increased fraction of bound water and tighter H-bonding in cross-linked matrices. By contrast, the CH_2_ overtone (~1180–1185 nm) remains nearly invariant, indicating that the collagen backbone is preserved and that the main changes arise from intermolecular interactions and microenvironmental polarity rather than gross chain alterations. Taken together, these spectral trends are coherent with the FT-IR indicators of cross-linking and with the monotonic decrease in cumulative drug release at higher GA content (Section 3.1 and Section 3.5).

### 3.3. Assessment of Hydrophilic Properties via Swelling Test

**Figure 4:**
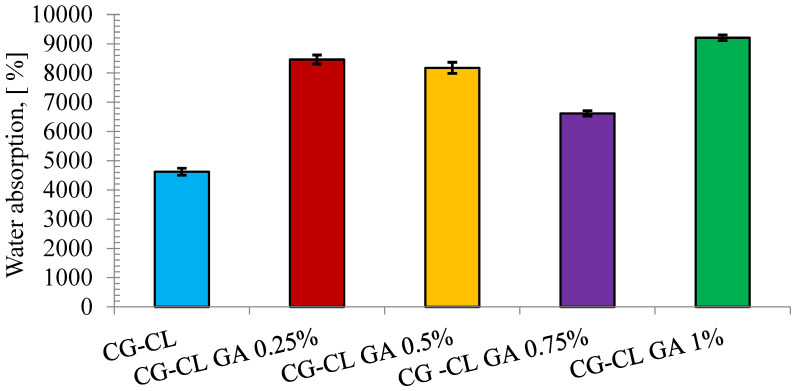
Water absorption (%) of CL-loaded collagen sponges as a function of GA content. Data are mean ± SD (*n* = 3).

To evaluate the water absorption capacity of the sponge containing collagen and chlortetracycline, but also of the sponges in which the cross-linking agent was added, the swelling test was performed (Figure 4). The collagen–chlortetracycline sponge exhibited high water absorption. When GA was introduced at low–intermediate levels, the swelling capacity did not significantly decrease; a slight upward shift was observed in some formulations. Our previous research studies on collagen sponges [10,40] also showed that water absorption is influenced by both the presence of the drug and the cross-linking agent, and even its concentration. In the case of collagen sponges in which antibiotics were incorporated, such as oxytetracycline hydrochloride (OTC) [10]—an antibiotic of the first generation tetracycline class as chlortetracycline, or chloramphenicol (CP) or doxycycline hydrochloride (DXC) [10,40]—antibiotics of the second generation tetracycline class, it was observed that sponges without GA absorb water, and by keeping the amount of antibiotic constant and using GA, a higher absorption capacity was observed for sponges with OTC or CP, and smaller in the case of collagen sponges with DXC. In a research study where collagen sponges were loaded with the anti-inflammatory active ingredient, ibuprofen (IB), the water absorption was very high, but the addition of GA significantly decreased the values [8]. Moreover, by using the same cross-linking agent, GA, an important component in maintaining the structural integrity of the sponges, ts continued to exhibit the ability to absorb water, but, depending on the drug used, this capacity decreased or increased slightly.

Notably, the difference between the 0.25% and 0.5% GA samples was minimal, with no clear trend indicating significant mechanical improvement within this concentration interval.

Although cross-linking generally constrains swelling, GA at low–intermediate levels likely stiffen the gel prior to freezing, limiting collapse during lyophilization and yielding a more open pore network; additionally, collagen–GA–chlortetracycline interactions increase bound water via hydrogen bonding. These factors rationalize why swelling did not markedly decrease in this window.

### 3.4. In Vitro Enzymatic Degradation Test

Considering that these chlortetracycline-loaded collagen sponges are prepared to be used in dental applications as drug delivery systems to prevent or treat local infections, they must exhibit good stability over time. For this purpose, in vitro enzymatic degradation was performed.

For the in vitro enzymatic degradation test, the initial weight of the discs was controlled and ranged between 0.140 g and 0.152 g. For each formulation, three separate release tests were conducted (*n* = 3).

The enzymatic degradation was performed using collagenase from *Clostridium histolyticum*, as it selectively targets collagen and allows controlled evaluation of matrix stability. While artificial saliva may offer a closer physiological context, the use of a specific collagen-degrading enzyme enabled us to isolate the effect of glutaraldehyde cross-linking on the structural resistance of the sponges.

Future studies should explore degradation profiles in artificial saliva or simulated oral fluids to better reflect conditions, especially considering the complex enzymatic environment present in the oral cavity.

**Figure 5:**
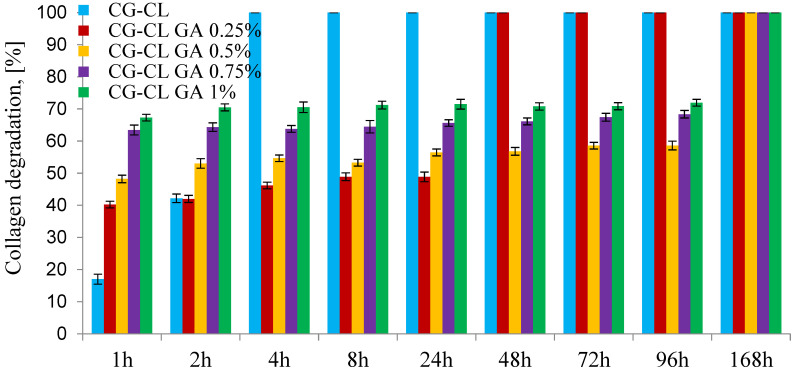
In vitro degradation of CL-loaded collagen sponges.

The results presented in Figure 5 show that the collagen sponge without cross-linking agent degrades completely after 4 h, but the sponges become stable when GA is used. The CG-CL GA 0.25% sponge degraded in the first 48 h, while the sponges with more than 0.5% GA degraded more slowly, the process continuing for up to 168 h. It was found that the addition of GA slowed down the degradation process, but the weight loss did not depend on the amount of GA in the case of samples in which the GA concentration was between 0.5% and 1%. Comparing these results with those obtained previously [8,10,40], the enzymatic degradation process proved to be slower in the case of antibiotics than anti-inflammatories.

Antibiotic-loaded sponges degraded more slowly than anti-inflammatory analogues. This likely reflects chlortetracycline’s polar, polyfunctional structure, which enhances hydrogen bonding with collagen and fosters secondary interactions within the GA-cross-linked network, thereby limiting collagenase access. In contrast, hydrophobic anti-inflammatory agents (e.g., ibuprofen) can weaken local H-bonding and create microdomains more accessible to enzymatic attack, consistent with our spectroscopic trends (Section 3.1 and Section 3.2).

### 3.5. In Vitro Kinetics Release of Chlortetracycline

For the in vitro release study, each specimen was trimmed from the freeze-dried sponge to fit the sandwich-device window. The mass of the discs was controlled and ranged between 0.140 g and 0.152 g. All release tests were performed in triplicate (*n* = 3) for each formulation.

**Figure 6:**
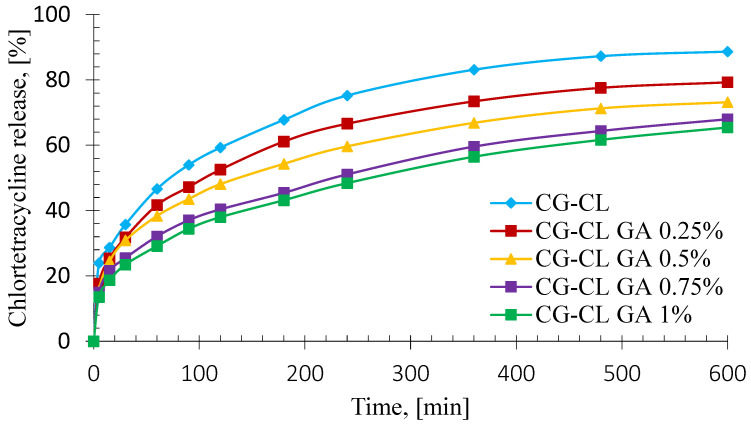
CL release as a function of time for collagen-based sponges. Data are expressed as mean ± standard deviation (*n* = 3).

The kinetic profiles corresponding to the controlled release of the active substance as a function of time, recorded for chlortetracycline-loaded collagen sponges with and without cross-linking agent, are presented in Figure 6.

Beyond illustrating cumulative release, the profile shape also provides insights into the release rate, highlighting an initial burst followed by a gradual slowdown—consistent with diffusion-controlled kinetics typical for collagen-based matrices. It is noticed that the shape of the release curve is relatively similar and does not depend on the amount of the cross-linking agent. This means that the antibiotic is released in a similar manner for any concentration of glutaraldehyde, but the percentage of antibiotic released depends inversely on the concentration of cross-linker.

From Figure 6, it can be noticed that the release kinetics of chlortetracycline from collagen-based sponges had a biphasic profile, with two different stages. The initial stage, also known as the” burst release” phase, corresponding to the first 60 min was characterized by a rapid and accelerated drug release. This drug behavior is likely caused by the dissolution of the antibiotic from surface-deposited particles on the sponges [62], combined with the rapid diffusion of chlortetracycline from the sponges due to their high swelling capacity upon contact with the aqueous release medium (Figure 4). After this rapid phase, a slower drug release stage took place for the next 10 h. This effect of the sustained chlortetracycline release could be due to entrapment of the antibiotic into the three-dimensional network of the collagen sponges, which caused a continuous delivery of the drug, with a continuous increase on the chlortetracycline released cumulative percentages [10]. These drug-release properties are advantageous for both the prevention and treatment of infections related to various dental procedures, where it is essential to rapidly rich a high concentration of the antibiotic at the affected oral area, followed by a sustained release to prevent further bacterial expansion [63].

The burst release effect varied from 29.17% (CG-CL GA 1%) to 46.65% (CG-CL), indicating that the increase of the GA concentration at the maximum level led to a less pronounced burst release effect, being 1.60 times smaller than the burst release recorded for the uncross-linked sponge.

After 10 h, the chlortetracycline cumulative percentages ranged from 65.48% for CG-CL GA 1% sample to 88.69% for CG-CL sample. Comparing the uncross-linked one sample with the cross-linked ones, it can be observed that the cumulative drug release for the uncross-linked was 1.12 times higher than the value of the sponge with 0.25% GA, 1.21 times higher than the value of the sponge with 0.5% GA, 1.30 times higher than the value of the sponge with 0.75% GA, respectively 1.35 times higher than the value of the sponge with 1% GA. This behavior is due to the strong cross-linking bonds determined by the increase in the amount of glutaraldehyde, which implies a slower release of the antibiotic from the tested matrices. These high values of the antibiotic cumulative percentages are desirable for the prevention and the treatment of the infections that can occur after a dental procedure.

To establish the mechanism of the antibiotic delivery from the chlortetracycline-loaded collagen sponges, two kinetic models were applied: Higuchi and Power law Models (Equation (3)). The performance of every kinetic model was assessed by two significant statistical parameters: the correlation coefficient (R) and the adjusted R squared (adj. R^2^) determination coefficient. The values of both parameters are listed in Table 3, alongside the kinetic descriptors of the Power law model.

As is shown in Table 3, the highest values for both R and adjusted R^2^ parameters were registered for the Power law model, indicating that the kinetic data were best fitted by this mathematical model. The R values varied from 0.9960 to 0.9994, while the values of the adjusted R^2^ ranged from 0.9910 to 0.9992. The values for both kinetic parameters were higher than the values of the same descriptors recorded for the Higuchi model, suggesting that the Power law model is suitable for the description of the drug release mechanism from the collagen-based sponges. To extend the statistical evaluation of the drug release kinetics, the Akaike Information Criterion (AIC) was performed to assess the accuracy and the precision of the models. Because the experimental data were limited, the analysis was based on the corrected Akaike Information Criterion (AIC_c_), which furnishes a more robust estimation of the mathematical model fit [63]. The kinetic model deemed most statistically representative of the drug delivery process is the one with the lowest AIC_c_ values [64].

The Power law model was found to be the most appropriate for describing the drug release behavior, as it provided the best statistical fit among the tested models. This conclusion was based on both correlation coefficients (R and adjusted R^2^) and on the corrected AICc, a metric that balances goodness-of-fit with model complexity. Lower AICc values indicate a better-fitting model with fewer risks of overfitting, which in our case consistently favored the Power law approach over the Higuchi model. Furthermore, the difference of the AIC_c_ values between both models was greater than 2 [65], suggesting that the Power law model had a superior statistical performance to describe the drug release mechanism from the sponges.

Considering the disc geometry, the fitted exponents (*n* = 0.29–0.34) are consistent with diffusion-dominated (quasi-Fickian) release with a minor contribution from matrix relaxation. The biphasic profile reflects (i) an initial burst (~0–60 min) driven by rapid water uptake and dissolution/diffusion of surface-near drug (Section 3.3), followed by (ii) a sustained stage governed by diffusion through a progressively hydrated, more tortuous network as GA increases, in line with the decrease of k with GA (Table 3). Because the release medium was PBS (no collagenase), longer-time contributions are attributed to polymer relaxation/pore opening rather than enzymatic erosion. This interpretation is also coherent with the slower enzymatic degradation of CL-loaded matrices (Section 3.4). Although the drug-to-polymer ratio was maintained during sample preparation, the potential impact of glutaraldehyde concentration on the encapsulation efficiency of chlortetracycline was not investigated. Given that higher cross-linking densities may limit drug entrapment, this aspect remains an open question. Future research should address the relationship between GA content and loading efficiency to optimize both structural integrity and drug availability.

### 3.6. Evaluation of Antibacterial Activity

A detailed assessment of bacterial susceptibility to chlortetracycline was performed to establish the concentration-dependent antimicrobial response of each strain. Table 4 summarizes the susceptibility of the tested microorganisms to chlortetracycline across a concentration gradient. *E. coli* exhibited resistance starting at 100 µg/mL, while *S. aureus* showed slightly greater susceptibility, with resistance observed from 50 µg/mL downward. *E. faecalis* proved the most tolerant, remaining susceptible down to 12.5 µg/mL.

Interval MICs (Table 4) confirmed the susceptibility ranking (*S. aureus* > *E. coli* > *E. faecalis*).

MICs are reported as interval estimates based on twofold dilution steps; full outcomes are provided in Table S1 (Supplementary Materials).

**Figure 7:**
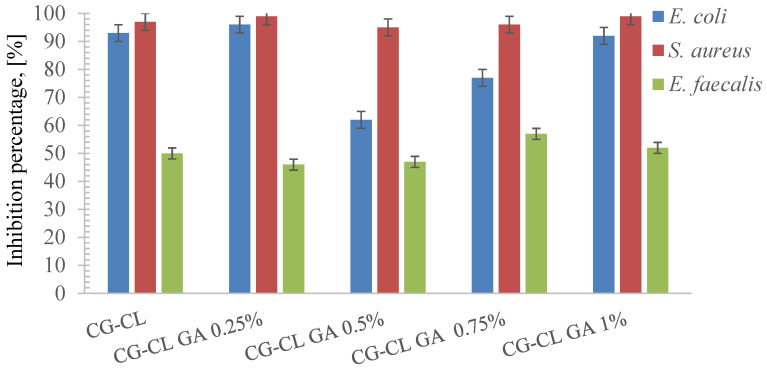
Antibacterial activity of CL-loaded collagen sponges against *E. coli*, *S. aureus*, and *E. faecalis*. Data are expressed as mean ± standard deviation (*n* = 3). Statistical differences between formulations were assessed using one-way ANOVA followed by Tukey’s post hoc test (*p* < 0.05). Significant differences, particularly for *E. faecalis*, are described in the main text.

As shown in Figure 7, *S. aureus* exhibited the highest inhibition percentages across all CG-CL formulations, followed by *E. coli*, while *E. faecalis* remained the least susceptible strain.

The inhibition remained relatively stable regardless of GA cross-linking, suggesting that *E. coli* is highly susceptible to chlortetracycline, and matrix density has a minimal impact on its release efficiency against this strain. The chlortetracycline-loaded collagen sponges demonstrated high inhibition percentages across all tested formulations, with values exceeding 90% in most samples (Figure 7).

The antibacterial performance of the chlortetracycline-loaded collagen sponges varied depending on the degree of cross-linking, offering valuable insight into how matrix structure influences bioactivity. The non-crosslinked sponge (CG-CL) consistently showed the strongest inhibition across all tested strains, confirming that minimal structural barriers favor rapid and effective drug diffusion. When 0.25% glutaraldehyde was introduced, the antimicrobial effect remained high, with only a slight decrease compared to the control, suggesting that at this level, the matrix still allows sufficient release of the antibiotic while gaining in structural integrity. As the GA concentration increased to 0.5% and 0.75%, a more visible decline in antibacterial activity was observed, particularly against *E. faecalis*, which appeared more affected by the reduced availability of chlortetracycline. At the highest cross-linking degree (1% GA), the inhibition rates were the lowest among all formulations, indicating that excessive reticulation can significantly hinder antibiotic release. Despite this, even the most cross-linked sponges retained a measurable antimicrobial effect, which demonstrates that the incorporated drug remains partially available. Overall, the results point to a clear trade-off: while higher cross-linking enhances sponge stability, it gradually limits antibacterial efficacy. Among the tested variants, the sponge with 0.25% GA stands out as the most balanced, preserving a strong antimicrobial profile while improving structural durability—an essential aspect for biomedical use in dental treatments. This reflects a design trade-off between achieving mechanical integrity through cross-linking and maintaining optimal drug release capacity. Future research could explore alternative cross-linkers such as genipin [66], a naturally derived compound with favorable biocompatibility and slower degradation profiles. Given its potential, part of our research group is currently investigating genipin-based systems for biomedical applications, with a patent application in progress. Other options include carbodiimide-based agents, offering tunable cross-linking densities, or the use of composite scaffolds incorporating polysaccharides or inorganic fillers to refine degradation behavior and therapeutic delivery.

*S. aureus* inhibition remains effectively maintained, confirming chlortetracycline’s strong efficacy against Gram-positive cocci even in more compact matrices. Slight variations were observed in inhibition levels across samples, ranging between 95% and 99%. While overall antibacterial activity remained high, the slightly lower values in samples with higher GA content suggest a minor diffusion barrier effect.

Among the tested strains, *E. faecalis* showed the most variation in inhibition efficiency, with values ranging between 46% and 57%. These lower values indicate partial resistance or reduced release of the antibiotic from the collagen matrix, particularly in samples with higher GA content. This confirms the need for careful formulation design when targeting persistent endodontic pathogens like *E. faecalis*. This observation was statistically supported by ANOVA and Tukey’s test (*p* < 0.05), confirming that the inhibition differences between formulations, especially for *E. faecalis*, were significant.

Beyond reduced local release, species-specific traits of *E. faecalis* can further limit chlortetracycline efficacy. Efflux systems and ribosomal protection proteins are known tetracycline resistance determinants in enterococci [67,68], raising the apparent MIC by lowering intracellular target engagement. In addition, the thick peptidoglycan of Gram-positive cells and the rapid formation of biofilms reduce antibiotic penetration and shift metabolism toward more tolerant states [69,70]. Within our cross-linked collagen network, higher GA increases path tortuosity and decreases effective diffusivity, which can lead to sub-MIC concentrations at the material–bacteria interface for this more tolerant species. These considerations align with our MIC intervals—*E. faecalis* (6.25–12.5 µg/mL) > *E. coli* > *S. aureus* (Table 4)—and with the “Proposed antibacterial mechanism” subsection, together supporting the observed susceptibility ranking.

The reduction in antimicrobial efficacy observed with increasing glutaraldehyde content can be attributed to several interrelated factors: increased cross-linking results in denser collagen matrices, restricting chlortetracycline diffusion; high GA content may immobilize the antibiotic within the network, reducing its availability for microbial inhibition. Therefore, the highest antimicrobial activity was observed in samples without cross-linking, indicating that optimal release correlates with minimal structural restriction. This reflects a design trade-off between mechanical stability and drug release efficiency, essential for dental biomaterials targeting microbial infections.

The inhibition graphs show the in vitro antibacterial activity of chlortetracycline-loaded collagen sponges with varying glutaraldehyde cross-linking levels. For *E. coli*, inhibition remained consistently high (>90%) across all formulations, demonstrating strong susceptibility and effective antibiotic release. *S. aureus* showed slightly more variation, with inhibiting values between 95% and 99%, suggesting a mild diffusion limitation in more cross-linked samples. *Enterococcus faecalis*, on the other hand, exhibited the lowest inhibition rates, between 46% and 57%. This reduced activity is likely due to a combination of moderate resistance and reduced release of chlortetracycline from the denser collagen matrix. These findings confirm the importance of considering both microbial susceptibility and matrix permeability when designing antimicrobial materials.

The observed trends can be attributed to multiple interrelated factors. Higher cross-linking reduces matrix porosity, thereby restricting drug diffusion as was observed in kinetic profiles corresponding to the controlled release of chlortetracycline from sponges. This, in turn, impairs the release of the antibiotic, especially in the case of persistent pathogens like *E. faecalis*. Additionally, the structural differences among the tested strains affect their response: Gram-negative bacteria such as *E. coli* are generally more permeable, while Gram-positive bacteria have thicker cell walls and more complex resistance mechanisms.

The proposed antibacterial mechanism outlines the sequence through which chlortetracycline, embedded in a collagen matrix, exerts its antimicrobial effect against bacterial pathogens.

(a) Drug incorporation and release

Chlortetracycline is loaded into the porous structure of a collagen sponge and upon exposure to an aqueous environment, the matrix swells and facilitates diffusion-controlled release of the antibiotic [10].

(b) Interaction with Gram-Positive bacteria (e.g., *S. aureus*, *E. faecalis*)

The antibiotic crosses the thick peptidoglycan layer characteristic of Gram-positive cells. It binds to the 30S ribosomal subunit, inhibiting protein synthesis. In *E. faecalis*, reduced susceptibility may be due to efflux pumps or biofilm formation, which physically restrict antibiotic access [71,72].

(c) Interaction with Gram-Negative bacteria (e.g., *E. coli*)

In Gram-negative bacteria, chlortetracycline passes through porin channels in the outer membrane. Once in the periplasm, it acts similarly by blocking translation at the ribosomal level. Because *E. coli* has thinner cell walls, the diffusion is faster, explaining the high inhibition percentages observed [73,74].

Increasing GA concentration reduces matrix porosity, slowing release kinetics. This leads to a lower bioavailability of the antibiotic, especially affecting persistent strains like *E. faecalis*.

The success of collagen-based antibiotic delivery relies not only on bacterial susceptibility, but also on the physico-chemical compatibility between the matrix and the drug, and the permeability characteristics of different bacterial cell envelopes (G(+) vs. G(−)). Thus, although the MIC table suggests high susceptibility of *E. faecalis*, its inhibition performance in sponge format is lower. This emphasizes the critical role of delivery system architecture in controlling therapeutic outcomes.

An important next step for the clinical applicability of our collagen sponges is the evaluation of cytotoxicity. Quantitative tests such as the MTT assay are recommended by ISO 10993-5 and are widely employed due to their reproducibility and objectivity [75,76]. In the dental biomaterials field, methods like MTT, LDH, or WST-1 are routinely used to assess cell viability in a dose- and time-dependent manner [77,78]. To place these biological findings in the context of drug transport, we further outline how cross-linking modulates diffusion within the collagen network. Mechanistically, the diffusion–stability trade-off described above can be rationalized as follows.

At increasing GA, the higher cross-link density reduces mesh size and free volume, while raising path tortuosity; together with stronger chlortetracycline–collagen/GA interactions (additional H-bonding sites), these effects decrease the effective diffusivity of the drug. The initial burst is driven by rapid water ingress and release of surface-near drug; the subsequent stage is controlled by diffusion through a progressively hydrated, denser network, consistent with the monotonic drop of k as GA increases and the quasi-Fickian exponents (*n* = 0.29–0.34). Spectroscopic indicators of increased bound water/H-bonding (Section 3.1 and Section 3.2) and the higher enzymatic stability corroborate this transport-limited regime. Within this framework, 0.25% GA provides sufficient structural stabilization while preserving a pore structure that still supports therapeutically relevant release.

To integrate physicochemical and biological results, we summarize how GA-driven structural changes propagate through swelling, stability, and transport to antibacterial outcomes.

Glutaraldehyde (GA) cross-linking modulates the full structure–property–function chain of the sponges. At the molecular level (Section 3.1 and Section 3.2), FT-IR and UV–VIS–NIR indicate strengthened collagen cross-linking and increased hydrogen bonding/bound water without denaturation. These changes shape the bulk response: hydrophilicity does not significantly decrease at low–intermediate GA (Section 3.3), and enzymatic stability increases (Section 3.4). The tighter, more H-bonded network lowers effective diffusivity and increases tortuosity, which is reflected in diffusion-dominated release (Section 3.5; *n* = 0.29–0.34 with k decreasing as GA increases). Consequently, higher GA can yield sub-MIC exposure at the material–bacteria interface and attenuate antimicrobial performance, whereas 0.25% GA provides a practical balance between structural stability and therapeutically relevant release (Section 3.6; see Table 4; Figure 7).

The observed strain-dependent responses highlight a crucial interplay between drug release dynamics and microbial susceptibility. While both *E. coli* and *S. aureus* maintained high inhibition percentages (>90%) even at 1% GA, *E. faecalis* showed a pronounced reduction (46–57%), reflecting its higher MIC, thick peptidoglycan wall, and ability to form biofilms [27,28,29,30,69,70], which collectively increase the threshold antibiotic concentration required for effective inhibition. In addition, efflux pumps and ribosomal protection proteins further limit intracellular tetracycline accumulation in enterococci [67,68]. Consequently, the reduced cumulative release from highly cross-linked matrices can fall below effective levels against *E. faecalis*, while still exceeding inhibitory thresholds for *E. coli* and *S. aureus* [71,72,73]. Clinically, this suggests that the design trade-off between matrix stability and antimicrobial efficacy is especially critical in endodontic infections dominated by *E. faecalis*. Formulations with moderate cross-linking (e.g., 0.25% GA) therefore provide the best balance, maintaining sufficient release to suppress resistant pathogens while ensuring structural durability.

## 4. Conclusions

Taken together, our data establish a clear design trade-off: increasing GA cross-linking strengthens the collagen network (greater structural/enzymatic stability) but progressively lowers the effective diffusivity of chlortetracycline and attenuates antibacterial performance. Within this continuum, the 0.25% GA formulation consistently offered the best balance, preserving a rapid initial release followed by sustained delivery up to 10 h and retaining strong antibacterial activity, while gaining stability versus the non-crosslinked control. In practical terms for dental biomaterials, this identifies 0.25% GA as the lead formulation for further development and provides a simple design rule: employ modest cross-linking to stabilize the matrix without compromising drug availability and avoid higher degrees (≥0.5% GA) that markedly diminish efficacy, particularly against *E. faecalis*.

For dental applications, achieving a balance between mechanical integrity and effective antimicrobial action is essential. The results highlight the importance of optimizing cross-linking conditions to ensure both durability and therapeutic efficacy of collagen-based drug delivery systems. A critical limitation of this study is the absence of cell viability data. Future research should include MTT or equivalent cytotoxicity assays, which are considered the standard for the safety assessment of biomaterials, especially in dental applications. Future in vivo studies should also investigate host tissue compatibility, potential immune and inflammatory responses, and long-term biocompatibility, which are essential aspects for clinical translation of these materials.

From a clinical perspective, the 0.25% GA formulation emerges as the most promising candidate, offering sufficient stability for handling dental procedures while maintaining strong antimicrobial efficacy. This balance is particularly relevant for applications such as tooth extractions or endodontic treatments, where preventing colonization by persistent pathogens like *E. faecalis* is critical. Future investigations should extend beyond cytotoxicity and in vivo animal models to clinical validation in complex oral environments, accounting for fluctuating pH, salivary flow, enzymatic activity, and the presence of polymicrobial biofilms.

## Figures and Tables

**Table 1:** Spectral data for chlortetracycline-loaded collagen sponges.

Sponge	A_OH_/A_I_	A_I_/A_OH_	Δυ
CG-CL	0.4557	2.1942	85
CG-CL GA 0.25%	0.4516	2.2142	82
CG-CL GA 0.5%	0.4489	2.2274	85
CG-CL GA 0.75%	0.4719	2.1191	85
CG-CL GA 1%	0.4441	2.2518	86

**Table 2:** UV–VIS–NIR band assignments and band positions (nm) for CL-loaded collagen sponges; small red shifts of OH-related bands with GA indicate increased bound water/H-bonding, while the near-constant CH_2_ overtone suggests an intact collagen backbone.

Band			λ, [nm]		
	CG-CL	CG-CL GA 0.25%	CG-CL GA 0.5%	CG-CL GA 0.75%	CG-CL GA 1%
-CO-NH-	293	304	291	296	307
ν_CH2_	1184	1185	1180	1181	1181
ν_OH ass_	1497	1499	1502	1502	1503
δ_O-H_	1944	1948	1946	1948	1950

**Table 3:** Statistical parameters of the Higuchi and Power law models; kinetic parameters specific to the Power law mathematical model; cumulative chlortetracycline release percentages.

Sponge	Higuchi Model			Power Law Model			Kinetic Constant, k (1/min^n^)	Release Exponent, *n*	CL Released (%)
	R	Adj R^2^	AIC_c_	R	Adj R^2^	AIC_c_			
CG-CL	0.9675	0.9297	−55.52	0.9958	0.9910	−80.19	0.142	0.29	88.69
CG-CL GA 0.25%	0.9688	0.9326	−58.46	0.9960	0.9914	−83.12	0.120	0.30	79.31
CG-CL GA 0.5%	0.9702	0.9355	−61.35	0.9978	0.9954	−93.01	0.112	0.30	73.22
CG-CL GA 0.75%	0.9838	0.9647	−71.05	0.9994	0.9988	−111.7	0.085	0.32	67.99
CG-CL GA 1%	0.9879	0.9736	−75.24	0.9992	0.9992	−117.3	0.073	0.34	65.48

**Table 4:** Minimum inhibitory concentration (MIC) intervals for CL (µg/mL) against the tested microorganisms.

Microorganism	MIC (µg/mL)
*Escherichia coli*	(100, 200]
*Staphylococcus aureus*	(50, 100]
*Enterococcus faecalis*	(6.25, 12.5]

## Data Availability

The original contributions presented in this study are included in the article/Supplementary Material. Further inquiries can be directed to the corresponding author.

## Supplementary Materials

The following supporting information can be downloaded at: https://www.mdpi.com/article/10.3390/ma18174029/s1, Table S1: Susceptibility outcomes (S/R) across the chlortetracycline dilution series used to derive MIC intervals.

## Abbreviations

The following abbreviations are used in this manuscript:

*E. coli*	*Escherichia coli*
*E. faecalis*	*Enterococcus faecalis*
*S. aureus*	*Staphylococcus aureus*
GA	Glutaraldehyde
CG	Collagen Gel
CL	Chlortetracycline HCL
NaOH	Sodium Hydroxide
ATR	Attenuated Total Reflection
LB	Luria Bertani
MIC	Minimum inhibitory Concentration
OD	Optical density
SD	Standard Deviation
OTC	Oxytetracycline Hydrochloride
CP	Chloramphenicol
DXC	Doxycycline Hydrochloride
IB	Ibuprofen
R	Correlation Coefficient
AIC	Akaike Information Criterion
