# Supplementary material for: Physical–Chemical Assessment and Antimicrobial Activity of Chlortetracycline-Loaded Collagen Sponges
Source: Materials (Basel). 2025 Aug 28;18(17):4029. doi: 10.3390/ma18174029 (PMC12429324; doi:10.3390/ma18174029)
Supplement: Supplementary file 1 [file materials-18-04029-s001.zip › materials-3817834-supplementary.pdf]

Supplementary material

Physical-chemical assessment and antimicrobial activity of chlortetracycline-loaded collagen sponges

Graţiela Teodora Tihan [1], Camelia Ungureanu [1], Ileana Rău [1], Roxana Gabriela Zgârian [1,*], Răzvan Constantin Barbaresso [1], Mădălina Georgiana Albu Kaya [2], Cristina-Elena Dinu-Pîrvu [3,4] and Mihaela Violeta Ghica [3,4]

1 Faculty of Chemical Engineering and Biotechnology, Department of General Chemistry, National University of Science and Technology Politehnica of Bucharest, 1-7 Gheorghe Polizu Street,
011061 Bucharest, Romania; gratielatihan@yahoo.com (G.T.T.); ungureanucamelia@gmail.com (C.U.);
ileana.rau@upb.ro (I.R.); razvanb67@yahoo.com (R.C.B.)
2 Division of Leather and Footwear Research Institute, Department of Collagen, National Research and Development Institute for Textiles and Leather, 93 Ion Minulescu Str.,
031215 Bucharest, Romania; albu_mada@yahoo.com
3 Faculty of Pharmacy, Department of Physical and Colloidal Chemistry, "Carol Davila" University of Medicine and Pharmacy, 6 Traian Vuia Street, 020956 Bucharest, Romania; ecristinaparvu@yahoo.com (C.-E.D.-P.); mihaelaghica@yahoo.com (M.V.G.)
4 Innovative Therapeutic Structures Research and Development Center (InnoTher), "Carol Davila" University of Medicine and Pharmacy, 6 Traian Vuia street, 020956 Bucharest, Romania
* Correspondence: zgirianroxana@yahoo.com

Table S1. Susceptibility outcomes (S/R) across the chlortetracycline dilution series used to derive MIC intervals.

Microorganism	400 μg/mL	200 μg/mL	100 μg/mL	50 μg/mL	25 μg/mL	12.5 μg/mL	6.25 μg/mL	3.125 μg/mL	1.56 μg/mL	0.78 μg/mL	0.39 μg/mL	0.195 μg/mL
Escherichia coli	S	S	R	R	R	R	R	R	R	R	R	R
Staphylococcus aureus	S	S	S	R	R	R	R	R	R	R	R	R
Enterococcus faecalis	S	S	S	S	S	S	R	R	R	R	R	R

Abbreviations: S = susceptible (growth inhibited), R = resistant (growth not inhibited). Concentration steps: 400, 200, 100, 50, 25, 12.5, 6.25, 3.125, 1.56, 0.78, 0.39, 0.195 μg/mL. n = 3.
